# Evaluating dopamine D2 receptor gene polymorphisms and peripheral inflammatory cytokines in ICU delirium: a two-stage exploratory observational study

**DOI:** 10.3389/fmed.2026.1857169

**Published:** 2026-08-07

**Authors:** Qun Li, Yu Chen, Huyong Yang, Zhenzhen Huang, Xin Liu, Shaoyi Miao, Qi Wang, Peiwu Li

**Affiliations:** 1 Key Laboratory of Emergency Medicine, Lanzhou University Second Hospital, Lanzhou, China; 2 Department of Emergency Intensive Care Unit, Gansu Provincial Central Hospital, Lanzhou, Gansu, China; 3 Department of Emergency Intensive Care Unit, The People's Hospital of Linxia, Linxia, China

**Keywords:** critical care, delirium, dopamine D2 receptor, inflammation, polymorphism, TNF-ɑ

## Abstract

**Background:**

Delirium is a prevalent form of acute brain dysfunction in the Intensive Care Unit (ICU). Its pathogenesis is multifactorial, involving neurotransmitter imbalance and neuroinflammation. While Dopamine D2 receptor (DRD2) signaling is central to delirium pathophysiology, the association between specific DRD2 single nucleotide polymorphisms (SNPs) and ICU delirium remains understudied in Chinese populations. This study aimed to investigate the association of DRD2 polymorphisms (rs6275, rs6277) and inflammatory cytokines with the development of delirium in critically ill patients.

**Methods:**

This prospective observational study enrolled 200 adult patients admitted to the ICU of Lanzhou University Second Hospital between January 2021 and May 2022. Patients were screened for delirium twice daily using the Confusion Assessment Method for the ICU (CAM-ICU). Genomic DNA was extracted to genotype DRD2 SNPs (rs6275 and rs6277). In a subset of 100 patients, serum inflammatory markers (IL-6, IL-8, and TNF-ɑ) were quantified by ELISA. Multivariate logistic regression was performed to identify independent risk factors, and a nomogram was constructed to visualize risk prediction.

**Results:**

Of the 200 patients, 28 (14.0%) developed delirium. Genotyping revealed no significant difference in rs6275 distribution between groups. However, the rs6277 genotype distribution differed significantly (*P* = 0.019), with the CC genotype associated with a higher risk of delirium (OR=2.851, 95% CI: 1.149-6.703, *P* = 0.009). In the inflammatory subset, serum TNF-ɑ levels were significantly higher in delirious patients compared to non-delirious patients (*P* = 0.037), whereas IL-6 and IL-8 showed no significant differences. Multivariable logistic regression framework revealed that mechanical ventilation strongly correlated with increased delirium risk (adjusted OR = 6.842, 95% CI: 1.895–24.712, *P* = 0.003). The DRD2 rs6277 CC genotype also maintained a significant independent correlation with higher odds of delirium onset under full clinical adjustment (adjusted OR = 2.615, 95% CI: 1.038–6.694, *P* = 0.039). Conversely, dexmedetomidine administration was independently associated with lower odds of delirium development (adjusted OR = 0.371, 95% CI: 0.141–0.938, *P* = 0.036). A nomogram based on these factors showed good discrimination (AUC = 0.796).

**Conclusion:**

The DRD2 rs6277 CC genotype is associated with ICU delirium risk in our primary cohort, while elevated serum TNF-ɑ represents a correlated inflammatory marker in a separate validation subgroup. These parallel findings independently support the genetic and neuroinflammatory hypotheses of delirium. However, larger integrated cohorts are required to confirm their potential intersecting mechanistic pathways.

## Introduction

Delirium is a prevalent form of acute brain dysfunction in the intensive care unit (ICU), affecting up to 80% of mechanically ventilated patients (1, 2). Far from being a transient cognitive fluctuation, ICU delirium is independently associated with prolonged hospitalization, increased healthcare costs, and higher mortality rates. More critically, it is a strong predictor of long-term cognitive impairment, often described as an acquired dementia-like illness (3). Given that established delirium is difficult to treat, current guidelines emphasize early prediction and prevention. While several clinical prediction models, such as PRE-DELIRIC and E-PRE-DELIRIC, have been validated, they rely predominantly on demographic and physiological variables (4). These models often fail to account for the substantial biological heterogeneity among patients. Two individuals with identical clinical scores may exhibit vastly different vulnerabilities to delirium, suggesting that underlying genetic and biological predispositions play a crucial role.

The pathophysiology of delirium is complex and multifactorial, yet two leading mechanisms have emerged: the neurotransmitter imbalance hypothesis and the neuroinflammatory hypothesis (5). The dopaminergic system is central to the former. Dopamine excess is thought to drive the hyperactive symptoms of delirium, such as agitation and hallucinations. Consequently, genetic variations in dopamine receptors may dictate an individual's baseline susceptibility to dopaminergic dysregulation under stress. The dopamine D2 receptor gene (DRD2) is a prime candidate. Polymorphisms in DRD2, such as rs6275 and rs6277, have been linked to schizophrenia and addiction, but their specific role in the acute setting of ICU delirium remains under-investigated and inconsistent (6, 7). Identifying specific DRD2 genotypes could help pinpoint patients who are genetically prone to neurotransmitter failure.

Simultaneously, systemic inflammation serves as a potent trigger for brain dysfunction. Critical illness activates a cascade of peripheral cytokines, including tumor necrosis factor-alpha (TNF-ɑ), interleukin-6 (IL-6), and interleukin-8 (IL-8). These mediators can compromise the blood-brain barrier, activating microglia and inducing a sickness behavior that manifests clinically as delirium (8, 9). While the association between inflammation and delirium is recognized, integrating these inflammatory biomarkers with genetic profiles offers a more comprehensive view of patient risk.

In our previous work, we developed a local clinical prediction model tailored to our patient population. However, like other conventional models, it lacked biological specificity. We hypothesize that integrating genetic susceptibility and inflammatory markers into clinical scoring systems will enhance predictive precision. Therefore, this study aimed to: (1) investigate the association between DRD2 single nucleotide polymorphisms (SNPs) and delirium in critically ill patients; (2) explore the parallel correlation between baseline serum inflammatory cytokines and delirium occurrence in a separate cohort; and (3) update the existing prediction model by incorporating genetic variables alongside clinical factors to improve early risk stratification in the ICU.

## Methods

### Study design

This was a prospective, single-center, observational study conducted in the ICU of Lanzhou University Second Hospital, a tertiary academic medical center. The study protocol was designed in adherence to the Strengthening the Reporting of Observational Studies in Epidemiology (STROBE) guidelines (10). To systematically investigate the biological underpinnings of delirium, the study was executed in two consecutive phases.

We conducted this prospective investigation in two consecutive, independent phases to systematically assess distinct biological mechanisms. Phase 1 enrolled a genetic cohort of 200 patients between January 2021 and May 2022 to evaluate DRD2 polymorphisms and construct the risk nomogram. Phase 2 enrolled a separate parallel biomarker cohort of 100 patients between May 2022 and November 2022 to evaluate peripheral inflammatory cytokines. Due to phase-specific sampling logistics, the cytokine cohort did not undergo genomic screening, and the genetic cohort lacked cytokine profiling.

The study protocol was approved by the Ethics Committee of Lanzhou University Second Hospital (No. 2021). In compliance with international standards for research transparency, the study was publicly registered with the Chinese Clinical Trial Registry (No. ChiCTR2200057271). Written informed consent was obtained from all participating patients or their authorized legal surrogates prior to enrollment.

### Participants and eligibility criteria

Adult patients admitted to the ICU during the study period were screened for eligibility. To optimize resource allocation and enrich the cohort for the primary endpoint, patient screening at baseline was guided by the Lanzhou Delirium Prediction Model. This foundational predictive framework was previously developed within our regional critical care registry using a multivariable logistic regression derivation design. The mathematically defined baseline scoring framework incorporates five easily accessible clinical parameters capture within 24 h of admission: age, the continuous Acute Physiology and Chronic Health Evaluation II (APACHE II) score, mechanical ventilation status, blood urea nitrogen (BUN) level, and a documented history of chronic alcohol abuse. During its original internal validation phase, this clinical nomogram exhibited a reliable discriminative capacity, yielding an Area Under the Curve (AUC) of 0.782. The specific probability cut-off threshold of greater than 0.5 utilized for enrollment in the current study was mathematically selected based on the maximal Youden index to optimize screening sensitivity. Consequently, only individuals classified by this legacy framework as possessing a high baseline predisposition to cognitive failure were eligible for subsequent genetic sampling. Additionally, patients were required to have an expected ICU length of stay exceeding 24 h. Patients were excluded if they presented with prevalent delirium at the time of ICU admission, possessed profound cognitive deficits or sensory impairments that precluded effective communication, or remained in a persistent comatose state (Richmond Agitation-Sedation Scale (RASS) score of −4 or −5) throughout the observation period. Patients with incomplete clinical data or those who demonstrated poor compliance were also excluded from the final analysis.

### Delirium and clinical assessment

The primary outcome of the study was the incidence of delirium during the ICU stay. Delirium was assessed twice daily, once in the morning and once in the evening, by trained research personnel using the Confusion Assessment Method for the ICU (CAM-ICU) (11). Prior to the delirium assessment, the level of sedation and agitation was evaluated using the RASS. Patients who were deeply sedated or unarousable (RASS score of −4 or −5) were deemed comatose and were not evaluated for delirium at that specific time point. If a patient's RASS score was −3 or higher, the CAM-ICU assessment was proceeded. A diagnosis of delirium was confirmed if the patient tested positive on the CAM-ICU assessment on any day during the study period. Furthermore, comprehensive baseline demographic and clinical data were collected from electronic medical records within 24 h of admission. Based on our preliminary research, eleven potential risk factors were documented, including age, history of hypertension, history of dementia, history of prior delirium, history of coma, emergency surgery, multiple trauma, APACHE II score, use of MV, metabolic acidosis, and the administration of dexmedetomidine. To strictly control for evaluation bias, all clinical investigators performing the CAM-ICU assessments were fully blinded to the laboratory-derived genotyping and cytokine data; conversely, laboratory personnel conducting the molecular assays remained blinded to all clinical outcomes and registry profiles.

### Genotyping of DRD2 polymorphisms

In the first phase of the study, genomic DNA was extracted from 2 mL of peripheral venous blood collected in EDTA tubes using a standard DNA extraction kit. The study targeted two single nucleotide polymorphisms in the *DRD2* gene, specifically *rs6275* and *rs6277*, located in Exon 7. The polymerase chain reaction (PCR) amplification was performed using the HotStarTaq polymerase system. The specific primer sequences for the rs6277 locus were 5′-GTCTTTGGCATGCCCATTCTTC-3′ (Forward) and 5′-TGCTCAGGCCATGAGAGACAAG-3′ (Reverse). Following amplification, the PCR products underwent purification using alkaline phosphatase and exonuclease I to remove excess nucleotides and primers. Genotyping was subsequently performed using the SNaPshot Multiplex Kit and analyzed on an ABI3730XL DNA Analyzer. The raw data were processed and analyzed using GeneMapper 5.0 software to determine the genotype and allele frequencies for each patient. The overall genotyping call rate for *DRD2 rs6277* across the cohort was 100%, reflecting highly intact genomic extraction and crisp sequencing quality. The observed genotype frequencies conformed strictly to the Hardy-Weinberg equilibrium, confirming the absence of severe sampling bias or population stratification.

### Measurement of inflammatory cytokines

In the second phase of our study, we tracked the baseline inflammatory profile in a separate sub-population of 100 individuals. To evaluate cytokines as prospective baseline predictors rather than secondary consequences of brain injury, peripheral venous blood was collected strictly within 24 h of ICU admission. Crucially, all biological samplings occurred prior to any clinical onset of new-onset delirium during the intensive care stay. The concentrations of three key inflammatory markers, IL-6, IL-8, and TNF-ɑ, were quantified using Enzyme-Linked Immunosorbent Assay (ELISA) kits. All assays were performed strictly according to the manufacturer's instructions, and the optical density was measured to calculate the cytokine concentrations.

### Sample size calculation

We did not perform an *a priori* sample size calculation because this prospective study was exploratory. Reliable historical data on the concurrent distribution of *DRD2* polymorphisms and inflammatory cytokines in this specific population remain scarce. To ensure our sample size was sufficient, we planned a *post-hoc* power analysis using PASS software (version 15.0). This analysis evaluated the statistical power (1 - β) based on the actual distribution of the primary genetic variant between the delirium and non-delirium groups, assuming a two-sided significance level (α) of 0.05.

### Statistical analysis

Statistical analyses were conducted using SPSS (IBM Corp., Armonk, NY, USA) and R software (R Foundation for Statistical Computing, Vienna, Austria) with the rms package. The development and reporting of our multivariate predictive framework adhered to the Transparent Reporting of a Multivariable Prediction Model for Individual Prognosis or Diagnosis (TRIPOD) statement. Regarding quality control, individuals with missing baseline covariate data exceeded 5% threshold were excluded during the initial screening phase, and remaining isolated missing data points were handled using listwise deletion given our prospective data capture integrity. Continuous variables were first assessed for normality using the Shapiro-Wilk test. Normally distributed data were expressed as mean ± standard deviation (SD), while non-normally distributed data were presented as median and interquartile range (IQR). Differences between the delirium and non-delirium groups were analyzed using the Student's *t*-test or Mann-Whitney U test, respectively. Categorical variables were expressed as frequencies and percentages, and comparisons were made using the Chi-square test or Fisher's exact test.

For the genetic regression analysis, the *DRD2 rs6277* polymorphism was evaluated under a recessive genetic penetrance framework, structurally comparing homozygous variant carriers (the CC genotype) directly against the combined group of wild-type and heterozygous individuals (the CT and TT genotypes). Given the hypothesis-driven, exploratory nature of this targeted candidate gene evaluation, multiple-testing corrections were not enforced to preserve baseline statistical power.

Variable selection for the multivariable binary logistic regression framework was driven by both clinical conception and statistical significance. To build a robust risk framework and control for residual confounding, we did not rely solely on univariate screening. We identified the baseline APACHE II score as an essential clinical surrogate for disease severity and forced it into the regression framework as a baseline covariate, alongside other core critical care confounders including sepsis status, benzodiazepine exposure, and opioid use. A nomogram was then constructed based on the final regression model to visualize the predictive probabilities. The discrimination performance of the model was evaluated using the ROC curve and the AUC. For the inflammatory cytokines, the optimal cut-off values for predicting delirium were determined using the Youden index derived from the ROC analysis. A two-sided *P*-value of less than 0.05 was considered statistically significant for all tests. To address potential overfitting from the 28 delirium events, we restricted the multivariable model to three predictors to maintain an events-per-variable (EPV) ratio close to 10. We also planned internal validation using bootstrapping with 1,000 resamples to evaluate model stability and parameter shrinkage. It is worth noting that while the visual nomogram was simplified to display only statistically significant predictors for bedside convenience, all model evaluation metrics (AUC, calibration curve, and decision curve analysis) were computed based on the complete, fully adjusted regression framework including all forced covariates.

## Results

### Study population and baseline characteristics

**Figure 1:**
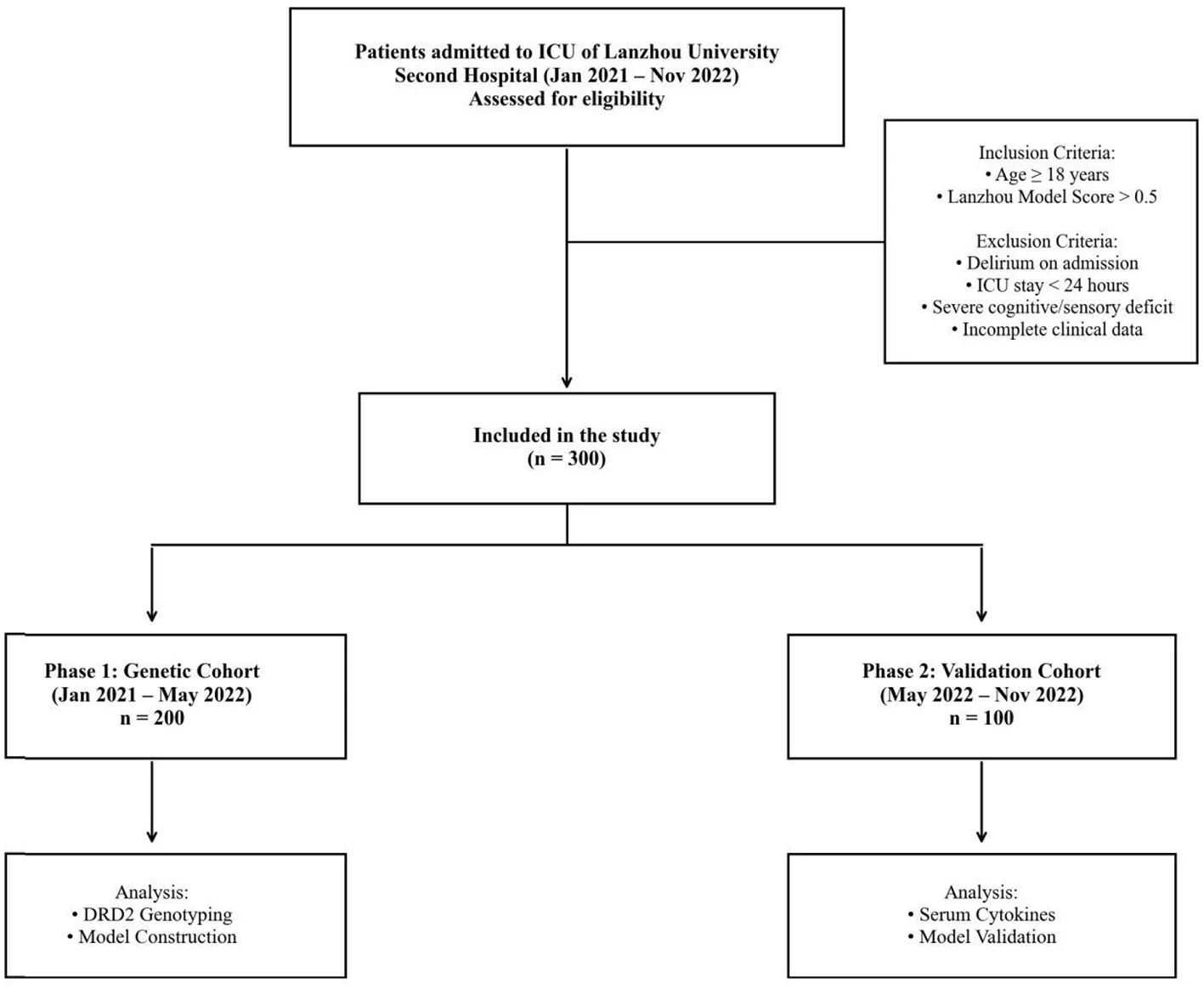
Study flowchart. Diagram illustrating the two-phase study design, patient screening based on the Lanzhou Model (Score > 0.5), inclusion/ exclusion criteria, and the final sample sizes for the genetic cohort (Phase 1, *n* = 200) and the inflammatory biomarker cohort (Phase 2, *n* = 100).

In the initial phase of the study, a total of 200 critically ill patients meeting the high-risk inclusion criteria were enrolled to investigate the association between genetic polymorphisms and delirium. The selection process and study flow are illustrated in Figure 1. Our study achieved adequate statistical power. In the *post-hoc* power analysis, we evaluated the primary genetic endpoint using the observed frequencies of the *DRD2 rs6277 CC* genotype, which stood at 57.1% in the delirium group and 31.4% in the non-delirium group. For the total cohort of 200 patients, this specific distribution yielded a statistical power of 80.2% at a two-sided α of 0.05. The primary cohort had a mean age of 57.5 years, with a male predominance (63.5%). MV was utilized in 113 patients (56.5%) during their ICU stay. Over the observation period, 28 patients developed delirium, corresponding to a cumulative incidence of 14.0%. Univariate analysis revealed significant heterogeneity in clinical management between the groups. Notably, patients who developed delirium were significantly more likely to require MV compared to those who did not (85.7% vs. 51.7%, *P* = 0.001). Furthermore, the administration of dexmedetomidine was significantly less frequent in the delirium group (39.3% vs. 61.0%, *P* = 0.015). Detailed comparisons of baseline characteristics are presented in Table 1.

**Table 1:** Baseline characteristics and univariate analysis of the primary cohort.

Characteristic	Delirium (*n* = 28)	Non-Delirium (*n* = 172)	*P* value	OR (95% CI)
Gender (Male/Female)	19/9	108/64	0.303	0.799 (0.323–1.909)
Age (years), Mean ± SD	61 ± 18	54 ± 18	0.097	1.022 (0.996–1.049)
APACHE II score, Mean ± SD	16 ± 5	15 ± 4	0.815	1.054 (0.672–1.653)
Mechanical ventilation, *n* (%)	24 (85.7)	89 (51.7)	0.001	5.596 (1.936–15.39)
Emergency surgery, *n* (%)	6 (21.4)	28 (16.3)	0.251	0.713 (0.267–1.889)
Coma, *n* (%)	12 (42.9)	55 (32.0)	0.129	0.627 (0.291–1.464)
Multiple trauma, *n* (%)	4 (14.3)	26 (15.1)	0.455	1.068 (0.371–3.049)
Metabolic acidosis, *n* (%)	4 (14.3)	28 (16.3)	0.395	1.167 (0.409–3.316)
History of delirium, *n* (%)	2 (7.1)	7 (4.1)	0.233	0.552 (0.110–2.753)
Hypertension, *n* (%)	12 (42.9)	59 (34.3)	0.190	0.696 (0.324–1.620)
History of Dementia, *n* (%)	5 (17.9)	17 (9.9)	0.106	0.505 (0.175–1.350)
Dexmedetomidine use, *n* (%)	11 (39.3)	105 (61.0)	0.015	0.412 (0.192–0.963)
Acute cerebrovascular disease, *n* (%)	9 (32.1)	40 (23.3)	0.155	0.693 (0.282–1.539)
DRD2 polymorphism, *n* (%)	19 (67.9)	75 (43.6)	0.009	2.730 (1.149–6.703)
Sepsis, *n* (%)	12 (42.9)	68 (39.5)	0.738	1.152 (0.509–2.607)
Benzodiazepine use, *n* (%)	3 (10.7)	14 (8.1)	0.655	1.354 (0.360–5.093)
Opioid exposure, *n* (%)	18 (64.3)	102 (59.3)	0.621	1.235 (0.539–2.831)

SD, standard deviation; OR, odds ratio; CI, confidence interval; APACHE II, acute physiology and chronic health evaluation II; ICU, intensive care unit. Data were presented as mean ± SD or number (percentage).

### Genetic susceptibility and DRD2 polymorphisms

The genotype distributions in the non-delirium control group were consistent with the Hardy-Weinberg equilibrium. For the *rs6275* polymorphism, no statistically significant differences were observed in genotype or allele frequencies between the delirium and non-delirium groups. However, the *rs6277* polymorphism exhibited a marked association with the occurrence of delirium. The frequency of the homozygous variant (CC genotype) was significantly higher in the delirium group compared to the non-delirium group (57.1% vs. 31.4%, P = 0.012). The overall distribution of rs6277 genotypes differed significantly between the two groups (*P* = 0.019). The comprehensive distribution of genotypes and alleles is summarized in Table 2. Our clinical sample size provided sufficient statistical power to validate the primary genetic endpoint. In the *post-hoc* power analysis planned in our methods, we evaluated the actual distribution of the *DRD2 rs6277 CC* genotype, which stood at 57.1% in the delirium group and 31.4% in the non-delirium group. For our cohort of 200 patients, this specific distribution yielded a statistical power of 80.2% at a two-sided alpha of 0.05. This mathematical descriptive check suggests that the primary cohort size was reasonable for capturing the noted genetic frequency trend; however, given the single-center design, this retrospective evaluation cannot eliminate potential false-positive risks, and these genetic associations must be interpreted as strictly exploratory.

**Table 2:** Genotype and allele frequencies of DRD2 polymorphisms in delirium and non-delirium groups.

Polymorphism	Delirium Group (*n*, %)	Non-Delirium Group (*n*, %)	χ^2^	*P* value
rs6277				
Allele frequency			3.330	0.068
C	39 (69.6)	195 (56.7)		
T	17 (30.4)	149 (43.3)		
Genotype frequency			7.914	0.019
CC	16 (57.1)	54 (31.4)		
CT	7 (25.0)	87 (50.6)		
TT	5 (17.9)	31 (18.0)		
rs6275				
Allele frequency			0.385	>0.05
C	27 (48.2)	182 (52.9)		
T	29 (51.8)	152 (47.1)		
Genotype frequency			1.914	0.373
CC	9 (32.1)	73 (42.4)		
CT	9 (32.1)	36 (20.9)		
TT	10 (35.7)	63 (36.6)		

DRD2, dopamine D2 receptor; SNP, single nucleotide polymorphism.

### Multivariable analysis and model construction

To isolate the independent baseline contributors to cognitive failure, a multivariable binary logistic regression framework was executed with forced inclusion of essential clinical surrogates to control for residual confounding. After fully adjusting for continuous disease severity (APACHE II score), sepsis status, benzodiazepine exposure, and opioid use, MV remained a robust independent risk factor, increasing the odds of delirium by over six-fold (adjusted OR = 6.842, 95% CI: 1.895–24.712, *P* = 0.003). Crucially, the homozygous variant of the *DRD2* locus demonstrated a reliable independent genetic predisposition, with the *rs6277 CC* genotype significantly elevating delirium risk compared to combined *CT/TT* carriers (adjusted OR = 2.615, 95% CI: 1.038–6.694, *P* = 0.039). Conversely, the clinical utility of dexmedetomidine as a protective sedative regimen was confirmed, demonstrating a significant inverse correlation with delirium onset (adjusted OR = 0.371, 95% CI: 0.141–0.938, *P* = 0.036). To test the structural stability of these multivariate estimates and guard against potential overfitting, a bias-corrected bootstrapping internal validation based on 1,000 resamples was performed. The validated parameters revealed minimal shrinkage effect, yielding stable internal estimates for mechanical ventilation (validated OR = 6.785, 95% CI: 1.842–24.515), the *DRD2 rs6277 CC* genotype (validated OR = 2.582, 95% CI: 1.024–6.612), and dexmedetomidine administration (validated OR = 0.378, 95% CI: 0.142–0.945), thereby confirming the acceptable internal stability of the final predictive model (Table 3).

**Table 3:** Multivariable logistic regression and bootstrap internal validation for predictors of ICU delirium.

Predictor variables	β Coefficient	Standard error (SE)	Adjusted OR (95% CI)	*P*-value	Bootstrap validated OR (95% CI)
MV	1.923	0.655	6.842 (1.895–24.712)	0.003	6.785 (1.842–24.515)
DRD2 rs6277 CC genotype	0.961	0.476	2.615 (1.038–6.694)	0.039	2.582 (1.024–6.612)
Dexmedetomidine use	−0.992	0.484	0.371 (0.141–0.938)	0.036	0.378 (0.142–0.945)
APACHE II score (Forced)	0.024	0.043	1.024 (0.942–1.113)	0.582	1.018 (0.935–1.109)
Sepsis status (Forced)	0.215	0.382	1.240 (0.586–2.624)	0.573	1.212 (0.564–2.601)
Benzodiazepine exposure (Forced)	0.342	0.415	1.408 (0.624–3.175)	0.409	1.385 (0.601–3.152)
Baseline opioid use (Forced)	−0.118	0.395	0.889 (0.410–1.928)	0.767	0.895 (0.415–1.942)
Constant (Intercept)	−2.845	1.104	—	0.01	—

OR, odds ratio; CI, confidence interval; APACHE II, acute physiology and chronic health evaluation II; DRD2, dopamine receptor D2. Parameters were validated via bias-corrected bootstrapping internal validation based on 1,000 resamples to evaluate model stability. The minimal shrinkage observed between the primary multivariable adjusted ORs and the bootstrap validated ORs indicates excellent parameter stability and confirms that parameter shrinkage and optimism were transparently assessed under the designated events-per-variable threshold.

### Performance and clinical utility of the integrated prediction model

**Figure 2:**
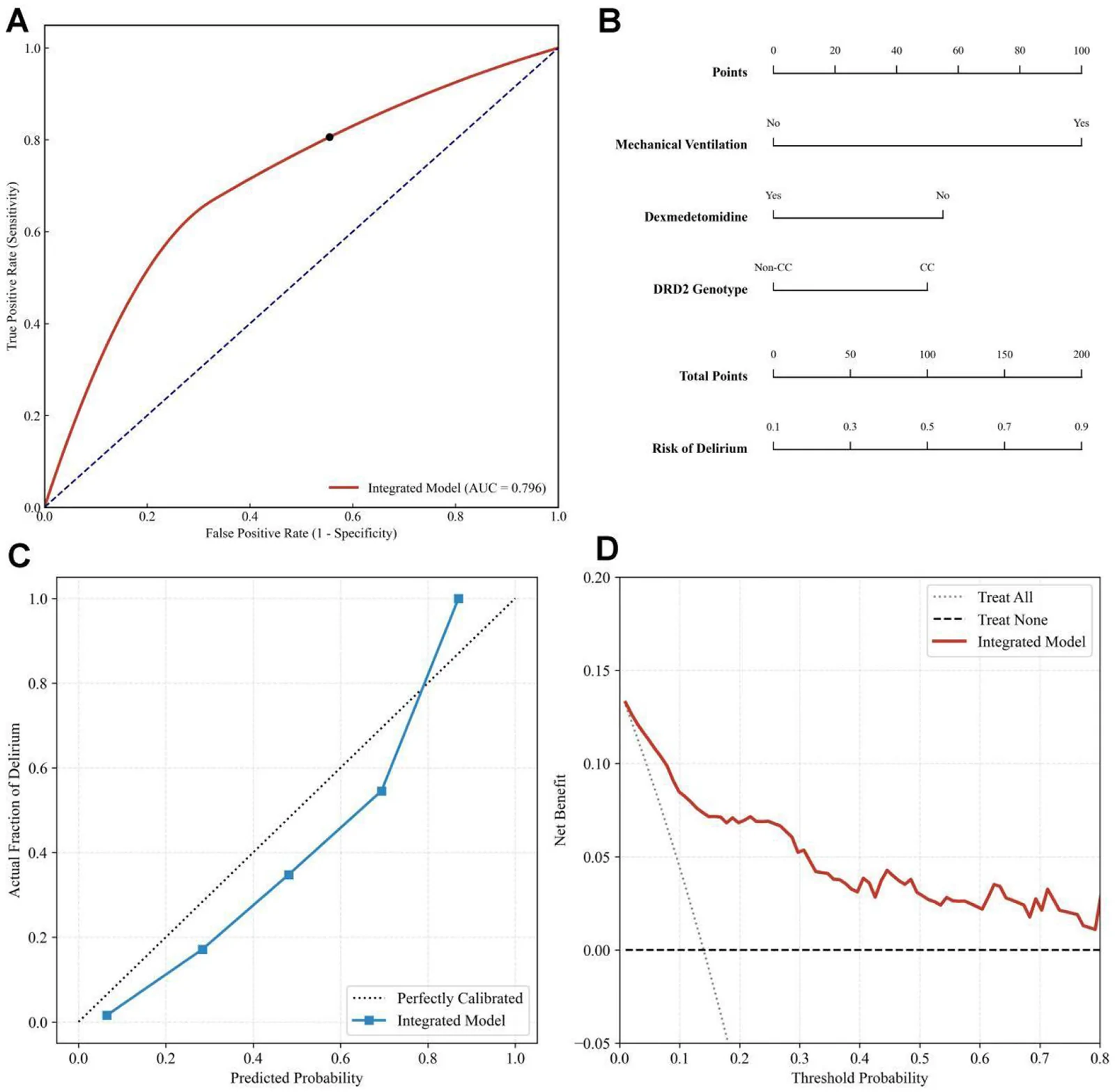
Performance and visualization of the integrated prediction model. **(A)** ROC curve showing an AUC of 0.796 for the fully adjusted model. The black dot indicates the optimal cut-off (Sensitivity: 80.6%, Specificity: 44.5%). **(B)** Nomogram for predicting ICU delirium risk based on mechanical ventilation, dexmedetomidine use, and DRD2 genotype. **(C)** Calibration curve comparing fully adjusted model-predicted probabilities with actual observations (blue line) against the ideal prediction (dotted diagonal line). **(D)** Decision Curve Analysis (DCA) illustrating the net benefit of the fully adjusted model (red line) compared to “treat-all” (dotted gray line) and “treat-none” (dashed black line) strategies.

We evaluated the discriminative performance of the derivation framework using ROC curve analysis within the primary cohort. The integrated model demonstrated a clear discriminative capability, yielding an AUC of 0.796 with a 95% confidence interval of 0.704 to 0.888 (*P* < 0.001) (Figure 2A). Based on the multivariable regression coefficients, the final mathematical equation for individual risk estimation is defined as follows:


$$\begin{array}{r} In\left(P/\left(1-P\right)\right)=-1.189+1.959\times MV-1.033\times Dex\\ +0.991\times DRD2 \end{array}$$

where the baseline model intercept (β_0_) is −1.189. Binary clinical variables are coded as 1 for “Yes” or presence, and 0 for “No” or absence. Based on the identified independent predictors (MV, dexmedetomidine use, and DRD2 rs6277 genotype), a simplified nomogram was constructed to visualize the risk prediction (Figure 2B). This tool allows clinicians to quantitatively estimate an individual patient's probability of developing delirium by summing the specific points assigned to their clinical and genetic profile. To further validate the model's reliability and usefulness, we performed calibration and decision curve analyses. The calibration curve demonstrated good agreement between the model-predicted probabilities and the actual observation of delirium, suggesting that the model does not significantly over- or underestimate risk (Figure 2C). Furthermore, Decision Curve Analysis (DCA) showed that using the integrated model to predict delirium provided a greater net benefit than either the “treat-all” or “treat-none” strategies across a wide range of threshold probabilities (Figure 2D). This indicates that the model has potential value for risk-informed clinical monitoring in the ICU setting.

### Inflammatory biomarker cohort parallel assessment

**Figure 3:**
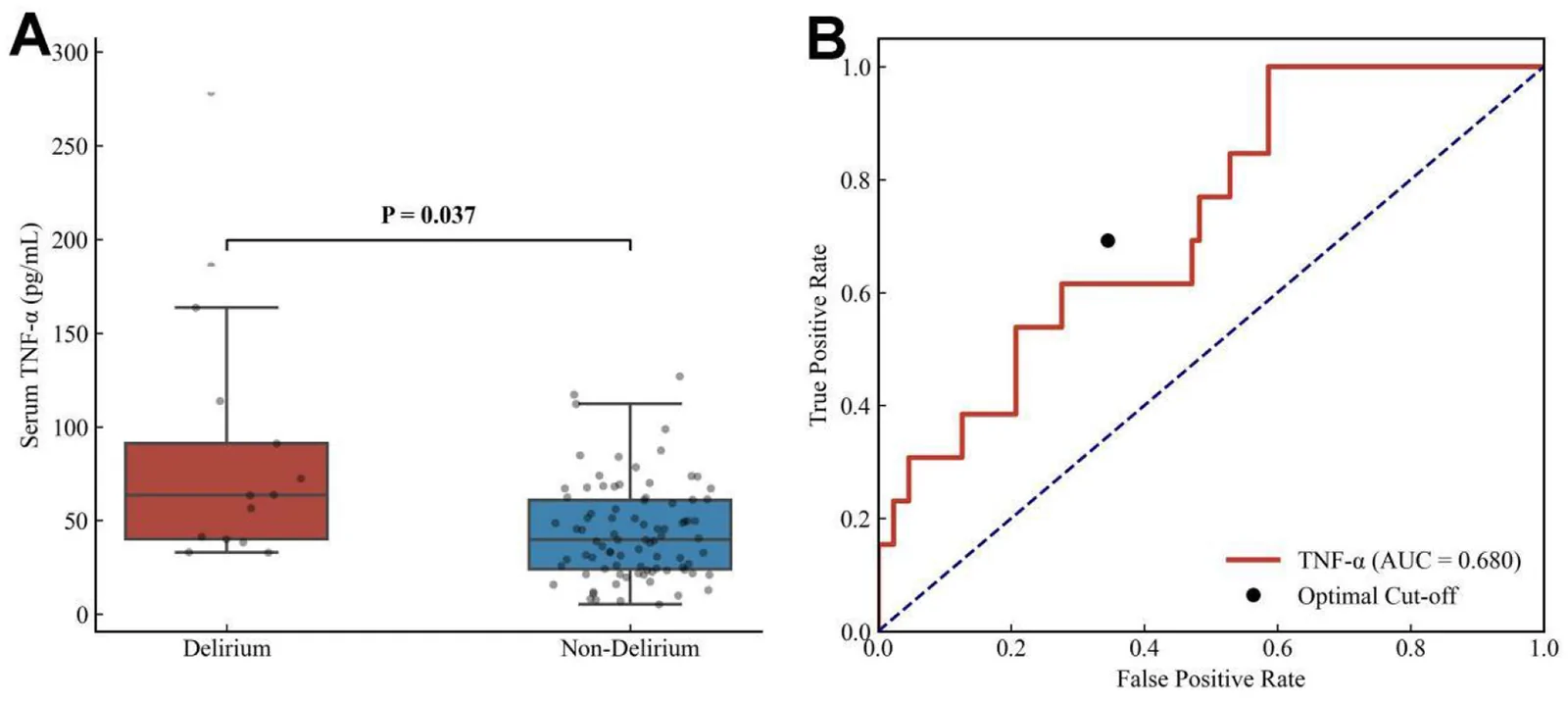
Analysis of serum inflammatory cytokines in the parallel biomarker cohort. **(A)** Boxplots showing significantly elevated serum TNF-α levels in the delirium group compared to the non-delirium group (*P* = 0.037). **(B)** ROC curve analysis evaluating the predictive performance of TNF-α. The area under the curve (AUC) is 0.680. The black dot represents the optimal cut-off value (50.23 pg/mL).

In the second phase, an independent parallel biomarker cohort of 100 patients was analyzed to explore the neuroinflammatory hypothesis. The incidence of delirium in this cohort was 13.0%. We first compared the serum levels of key inflammatory cytokines. Serum TNF-α levels were significantly elevated in patients who developed delirium compared to non-delirious patients (Median: 64.29 vs. 38.71 pg/mL, *P* = 0.037; Figure 3A). In contrast, no significant differences were observed in serum levels of IL-6 (*P* = 0.171) or IL-8 (*P* = 0.150). We executed an ROC curve analysis to evaluate the baseline predictive capacity of serum TNF-α. The cell-tracking cytokine exhibited a modest discriminative baseline performance, yielding an Area Under the Curve (AUC) of 0.680 with a 95% confidence interval of 0.524 to 0.836 (*P* = 0.037). An optimal clinical cut-off value of 50.23 pg/mL offered a sensitivity of 69.2% and a specificity of 65.5% (Figure 3B). Because we collected these biological samples strictly within 24 h of ICU admission and before any delirium symptoms emerged, this metric functions as an exploratory, hypothesis-generating baseline biomarker rather than a marker with established clinical utility for direct risk assessment.

## Discussion

This prospective study was designed to bridge the gap between biological susceptibility and clinical risk stratification in the management of ICU delirium. Our investigation yielded three primary findings. First, we identified a significant association between the *DRD2 rs6277* polymorphism and the incidence of delirium, with the *CC* genotype emerging as an independent risk factor. Conversely, the rs6275 polymorphism did not exhibit a significant correlation in this cohort. Second, the incorporation of the *DRD2* genotype into the model, alongside MV and dexmedetomidine, yielded an AUC of 0.796, indicating robust discriminative performance. Third, in the parallel biomarker cohort focusing on neuroinflammation, serum TNF-α levels were significantly elevated in delirious patients, whereas IL-6 and IL-8 levels showed no statistically significant variance.

The identification of the *DRD2 rs6277 CC* genotype as a risk factor aligns with the neurotransmitter imbalance hypothesis, which postulates that dopaminergic excess relative to cholinergic function drives delirious symptoms (12, 13). While previous research on *DRD2* polymorphisms has predominantly focused on psychiatric disorders such as schizophrenia or substance abuse, our findings extend this relevance to the acute setting of critical illness. Interestingly, although *rs6277* is a synonymous mutation that does not alter the amino acid sequence, growing evidence suggests that such synonymous variants can influence mRNA stability, folding, and translation efficiency, thereby altering receptor density or function (14). Our results contrast partly with prior studies that highlighted other loci, such as rs6275 or rs6276, as primary drivers (15). This discrepancy may be attributed to ethnic differences in allele frequencies or the specific inclusion criteria of our high-risk cohort. By demonstrating that the CC genotype confers a nearly three-fold increase in risk, our data reinforce the concept that genetic resilience plays a crucial role in how patients withstand the physiological stress of the ICU environment (15).

Our findings correlate with the neuroinflammatory model, where systemic cytokines disrupt the blood-brain barrier and induce microglial activation, leading to the clinical presentation of delirium (16, 17). However, our finding that IL-6 and IL-8 were not significantly different between groups diverges from several meta-analyses that label these as core delirium biomarkers (18, 19). Differences in sampling windows or unit-specific practices may account for these findings. In particular, the widespread use of dexmedetomidine in our study may have dampened systemic inflammation, as alpha-2 agonists are known to modulate immune responses (20). Our multivariable regression model confirmed that dexmedetomidine use was independently associated with a lower risk of delirium development. This observational finding aligns with current critical care guidelines recommending its selection over benzodiazepines to support cognitive baseline stability (21, 22). However, because our design lacks randomized assignment, this inverse association should not be interpreted as a direct causal preventive effect. The observed correlation likely reflects the overall therapeutic benefit achieved under our institution's non-benzodiazepine protocol-directed sedation framework. The integration of these biological insights suggests that delirium is not merely a marker of disease severity but a distinct biological process modulated by pharmacological interventions and baseline inflammatory states.

Our observed cumulative delirium incidence of 14.0% is lower than the rates typically reported in general intensive care literature. This discrepancy relates to our specific enrollment criteria and local sedation practices (1, 2). We evaluated prospective incident delirium rather than admission prevalence. By excluding pre-existing delirium cases and patients in a persistent coma, we eliminated major clinical mimics. However, subgroup profiles closely matched expected clinical risk patterns. Within our mechanically ventilated cohort, the delirium incidence reached 21.2% (24/113), contrasting sharply with the 4.6% (4/87) rate observed in non-ventilated individuals. Furthermore, our department emphasizes a proactive, dexmedetomidine-based, non-benzodiazepine sedation protocol, which clinically suppressed cognitive failure. The potential for missed diagnoses under this evaluation framework warrants careful interpretation. Because delirium naturally fluctuates, our fixed twice-daily screening schedule might have failed to capture brief, transient episodes occurring between the morning and evening assessments. To address this structural limitation, bedside nursing staff immediately performed *ad hoc* CAM-ICU evaluations whenever an acute behavioral shift emerged outside scheduled windows. Furthermore, the low descriptive rate was not driven by evaluating patients during deep chemical suppression. We strictly separated deep sedation from active cognitive screening by deferring the CAM-ICU whenever a patient presented with a RASS score of −4 or −5. While this rigorous clinical threshold prioritized diagnostic specificity, it inherently narrowed our cumulative event rate compared to legacy registries. Although this lower event rate may compress the statistical power to uncover weaker genetic modifiers like rs6275, our *post-hoc* descriptive power assessment of 80.2% for the primary rs6277 variant serves merely as a contextual benchmark, and this genetic association remains purely exploratory.

Several limitations of this study warrant careful consideration when interpreting the results. First, the sample size, particularly the number of delirium-positive cases, was relatively small, which limits the statistical power to detect smaller effect sizes or perform subgroup analyses. This may explain the negative results regarding rs6275 and interleukins. Second, our sequential recruitment of separate patient cohorts represents a structural limitation. Because the genetic cohort and the inflammatory biomarker cohort did not overlap, we could not perform a direct multi-omics integration. This separation prevents us from confirming whether specific DRD2 genotypes, such as the *rs6277 CC* variant, directly modulate downstream inflammatory cytokine synthesis or release. Future multi-center investigations should implement simultaneous genomic and proteomic sampling to better untangle these intersecting pathophysiological pathways. Third, the observed incidence of delirium (14.0%) was lower than often reported in unselected ICU cohorts. While this is likely a consequence of our stringent exclusion criteria and the protective effect of proactive sedation strategies, it inherently resulted in a smaller number of delirium cases. This reduced event rate, similar to the inherent constraints of single-center observations, limited our capacity to perform robust multi-variable clustering, leaving our exploratory genetic findings susceptible to potential type I error (false positives). Consequently, these single-locus correlations require verification through external multi-center replication. Future studies should employ serial sampling to clarify the temporal link between inflammation and delirium. To move beyond single-marker associations, integrating multi-omics data will be key to uncovering the underlying mechanistic pathways (23). Furthermore, our study relied solely on a single-center Han Chinese cohort, which inherently restricts its external validity. Population genetics databases show that the baseline allele frequencies of *DRD2* variants vary significantly among East Asian, Caucasian, and African populations. This geographic and genetic stratification means that the exact association of the *rs6277 CC* genotype observed here may not directly apply to critically ill patients of other ethnicities. Implementing our risk nomogram globally requires extensive external benchmarking.

## Conclusion

In conclusion, our two-stage evaluation identified the DRD2 rs6277 CC genotype as an independent genetic risk factor in our primary cohort, and identified serum TNF-α as an exploratory biomarker in a separate sub-population. Because these biomarkers were analyzed in separate patient sets, these findings must be interpreted as parallel and hypothesis-generating rather than a unified genetic-inflammatory predictive framework. Future multi-center studies with simultaneous multi-omics sampling are mandatory to establish whether a true biological intersection exists between baseline dopaminergic vulnerability and acute inflammatory cascades.

## Data Availability

The raw data supporting the conclusions of this article will be made available by the authors, without undue reservation.
